# Platelet kinetics after slow versus standard transfusions: A pilot study

**DOI:** 10.3109/03009734.2011.569588

**Published:** 2011-06-29

**Authors:** Abbas Habibi, Mohsen Esfandbod, Mohammad Hossein Ghafari, Patricia Khashayar, Atabak Najafi, Reza Shariat Moharari

**Affiliations:** ^1^Imam Khomeini Hospital, Tehran University of Medical Sciences, Tehran, Iran; ^2^Endocrinology and Metabolism Research Center, Tehran University of Medical Sciences, Tehran, Iran; ^3^Sina Hospital, Tehran University of Medical Sciences, Tehran, Iran

**Keywords:** Platelet decline, slow platelet transfusion, standard platelet transfusion

## Abstract

**Background:**

Platelet transfusion is required in the acute phase of some thrombocytopenic disorders in order to prevent potentially dangerous hemorrhages.The purpose of this study was to assess the increase in platelet count following a slow platelet transfusion.

**Methods:**

Patients suffering from thrombocytopenia due to various underlying diseases were enrolled in the prospective pilot feasibility trial and were randomly divided into two groups. Standard platelet transfusion was administered in one group, while slow transfusion was used in the other. The platelet count was examined at 1 hour, 24 hours, and 1 week following the transfusions.

**Results:**

Although the platelet count was higher following 1 hour after transfusion via the standard method, the count tended to be higher 1 week after the transfusion in the slow transfusion group. This difference, however, only turned out to be statistically significant amongst females.

**Conclusion:**

A therapy of slow platelet transfusion might be more effective for the prevention of platelet loss. Further studies will be required to strengthen this hypothesis.

## Introduction

Multiple platelet transfusions are commonly required in patients during the course of their treatment for a number of diseases. Alloimmunization to antigens expressed on platelets with refractoriness to platelet transfusion remains a major problem in these patients. In addition to the presence of allo-antibodies directed against platelet antigens, inadequate post-transfusion platelet counts can be due to a number of host-related factors, such as disseminated intravascular coagulation, splenomegaly, severe infection with high fever, drug-mediated antibodies, administration of amphotericin B, and by administration of platelets damaged or activated during collection or storage (1–3).

The incidence of human leukocyte antigen (HLA) alloimmunization and platelet refractoriness in patients receiving repeated transfusions of cellular components ranges between 20% and 70% (4). Although the mechanism of accelerated platelet destruction is not well understood, most studies have focused on non-immune host factors as the most common cause of refractoriness. The efficacy of transfusion of non-HLA matched platelet concentrates in these patients has been assessed in several studies; other studies have introduced alternatives to alleviate this problem and improve the outcome (2,3).

Standard transfusion pumps available in hospitals have provided an opportunity to control the transfusion rate without negative impact on the platelets for several years (5). Narvios et al. have introduced slow transfusion of platelet concentrates in patients with acute myelogenous leukemia and refractory thrombocytopenia as a feasible and effective alternative method (6).

Not many other studies have been conducted to appraise this hypothesis; moreover, available studies have been performed in small groups of patients with specific underlying diseases. This study compares the increases in platelet levels after standard and slow transfusion in patients with different underlying diseases that require platelet transfusions. In this paper, we present preliminary results of our on-going study.

## Materials and methods

### Design and setting

After approval of the institutional ethics board, this prospective pilot feasibility trial was conducted on consecutive thrombocytopenic patients referred to the Emergency Department of Imam Khomeini Hospital to receive platelets between January 2006 and January 2007. All patients with a platelet count of <10,000/μL and patients with platelet count between 10,000 and 50,000/μL with active bleeding attributed to platelet dysfunction or thrombocytopenia received a platelet transfusion. A platelet concentrate was prepared from whole blood of single donors (random donor platelets) by light centrifugation and preparation of platelet-rich plasma followed by re-centrifugation to prepare a single unit of concentrated platelets.

It should be noted that according to the setting of our hospital all patients were first screened in the emergency department. Then they were admitted to the department in case of acute and/or emergent problems. Some of them were later referred to a different department. The screening physician was informed to take a complete history of the patients admitted with thrombocytopenia, and in case of a platelet transfusion we were informed.

### Main outcomes

Demographic data including the patients' age, gender, and history of their previous transfusions were gathered. They were allocated to two groups using a computerized random number generation technique. In group 1, transfusion via slow platelet transfusion was performed: 6 units of platelets were transfused within 6 hours using transfusion pumps. In group 2, transfusions were performed via the standard method of transfusion: 6 units were transfused within 3 hours.

The patients' platelet counts were measured at four different intervals: before transfusion, 1 hour, 24 hours, and 1 week after transfusion.

### Statistics

Gathered data were entered in SPSS v. 13 and analyzed. Frequencies of the continuous variables were presented as mean and standard deviation. Student's *t*-test was used to compare the patients' age distribution and platelet count. Therefore, repeated measures test and the analyses of covariance (ANCOVAs) were used to examine the impact of a factor (time of transfusion) on a dependent variable (bone parameters) adjusted for differences in covariates (platelet count). *P* values less than 0.05 were considered statistically significant.

## Results

There were 56 patients, with 31 (55.4%) females and 25 (44.6%) males in this pilot study. It should be noted that two cases in the slow transfusion and one case in the standard transfusion group required re-transfusion; all three patients were excluded from the study.

The mean age of all patients was 38.9 ± 18.8 years. The patients were reported to have had the underlying disease for an average of 13.2 ± 10.5 months (ranging from 4 to 60 months); all patients had received multiple transfusions prior to participating in our study. There was no significant difference between the two groups regarding age, gender, and the base-line platelet levels. However, the mean disease duration was longer in the slow transfusion group (16.4 months versus 10.0 months; *P* value = 0.016).

Acute myelogenous leukemia (AML) and acute lymphocytic leukemia (ALL) were the most common underlying diseases in our patients (Table I).

**Table I.** Responses to treatment in relation to the patients' basic diagnoses.

	Slow transfusion		Standard transfusion	
Diagnosis	Frequency (%)	Ref. (*n*)	Frequency (%)	Ref. (*n*)
ALL	7 (53.8)	1	6 (46.2)	0
AML	5 (35.7)	1	9 (64.3)	1
Aplastic anemia	6 (50)	1	6 (50)	2
Pancytopenia	3 (60)	1	2 (40)	0
Lymphoma	6 (100)	1	0	0
Multiple myeloma	0	0	1 (100)	0
CML	0	0	4 (100)	1
Myeloproliferative dis.	1 (100)	0	0	0

Ref. = refractoriness (defined as cases with low platelet count 1 hour following transfusion).

Platelet counts increased, in both groups, 1 hour following transfusion (Table II); the increase was greater in the standard group. In contrast to the standard group, however, no decline was noted in the platelet count 1 week following slow transfusion. Repeated ANOVAs showed a significant difference between different time intervals for gender and transfusion type.

**Table II.** Platelet count (×10^9^/L) (in the two groups) following transfusion.

	Transfusion					
	Slow	Standard	*P* value	MD	CI 95%	
Prior to infusion	10.5 ± 5.1	14.0 ± 8.2	0.064	-3.44	-7.09	0.20
1 hour following infusion	30.9 ± 16.8	47.5 ± 29.0	0.011	-16.65	-29.30	-4.00
24 hours following infusion	35.0 ± 26.4	41.2 ± 29.6	0.423	-6.20	-21.63	9.22
1 week after infusion	48.5 ± 68.8	33.8 ± 21.9	0.355	14.76	-17.56	47.09

MD = mean difference; CI 95% = confidence interval 95%.

**Figure 1.**
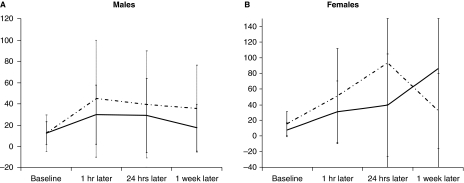
Platelet count (×10 ^9^/L) following standard (broken lines) or slow (full lines) transfusion in males (A) and females (B).

In repeated measure tests, significant interactions between time and gender and between time and transfusion type were found. Therefore, a regression analysis evaluating platelet count at different intervals (as the outcome) with base-line platelet, age, gender, and the relation between gender and transfusion type (as the predictors) was performed. The analysis showed that the type of transfusion significantly influences the platelet count within 1 hour after transfusion, whereas gender significantly influenced the count after 1 week of transfusion (Figure 1). Females responded better to transfusion compared with men (*P* < 0.05).

## Discussion

Several platelet transfusions in patients with continuous thrombocytopenia cause the immune system to produce antibodies against the platelet antigens. This leads to an alloimmunization phenomenon in which platelets become refractory to future transfusions (7,8).

With the introduction of new techniques that rapidly and reliably identify HLA antibodies, patients at risk for alloimmunization should be screened before or as early as possible in their course of platelet support. However, this method is not always sufficiently sensitive to detect all allo-antibodies present (9,10). As a result, recent studies have focused on achieving newer technologies to overcome the problem of alloimmunization. This situation, also known as refractory thrombocytopenia, is defined as abnormally low platelet count increments following two or more consecutive transfusions (11). However, the degree of abnormality depends on several factors. Transfusion dose (2–12 × 10^11^), patient blood volume, and the percent of platelets sequestered in an exchangeable splenic pool (normally about 33%) determine the expected peripheral platelet concentration increment (12). In one study, the velocity of the platelet transfusion appears to be a contributing factor to refractory thrombocytopenia (6).

Slow transfusion of platelet concentrates in thrombocytopenic patients has been tested in various case reports, all reported to be promising. In the study conducted by Narvios et al., three patients were treated for refractory thrombocytopenia in the course of acute myelogenous leukemia. Utilizing an alternative method of platelet transfusion, the platelet concentrate was infused slowly over 6 hours via a multichannel transfusion pump. The study showed that the platelet count increased significantly following this method; in addition, no side-effects were reported in these cases (6). Similarly, this study evaluated this method in a larger sample size and concluded that it could be considered as an acceptable method of platelet transfusion in thrombocytopenic patients.

Some earlier studies have shown both parous women and previously transfused patients to be at greater risk for alloimmunization to HLA, the most common cause of alloimmune transfusion refractoriness (13–15). However, the findings of this study conclude that women experienced a better response to slow platelet transfusion than did men. Unfortunately, the height and weight of the recipients were not measured, thus making it the major limitation of our study in this issue. Females are typically smaller than males, and the difference in responses to the experiment may be due to size. The difference between men's and women's platelet levels after slow platelet transfusions requires further studies.

## Conclusion

The present study showed that patients who received slow platelet transfusion were less prone to platelet decline after 1 week, even though the platelet count was higher 1 hour following transfusion via a standard method.

## References

[1] Wu KK, Thompson JS, Koepke JA, Hoak JC, Flink R (1976). Heterogeneity of antibody response to human platelet transfusion. J Clin Invest.

[2] Novotny VMJ (1999). Prevention and management of platelet transfusion refractoriness. Vox Sanguinis.

[3] Alcorta I, Pereira A, Ordinas A (1996). Clinical and laboratory factors associated with platelet transfusion refractoriness: a case-control study. Br J Haematol.

[4] McCullough J, Steeper TA, Connelly DP, Jackson B, Huntington S, Scott EP (1998). Platelet utilization in a university hospital. JAMA.

[5] Snyder EL, Rinder HM, Napychank P (1990). In vitro and in vivo evaluation of platelet transfusions administered through an electromechanical transfusion pump. Am J Clin Pathol.

[6] Narvios A, Reddy V, Martinez F, Lichtiger B (2005). Slow infusion of platelet: A possible alternative in the management of refractory thrombocytopenic patients. Am J Hematol.

[7] Murphy MF, Waters AH (1990). Platelet transfusions: The problem of refractoriness. Blood Rev.

[8] Shulman NR (1966). Immunological considerations attending platelet transfusion. Transfusion.

[9] Sandler SG (1997). Alloimmune refractoriness to platelet transfusions. Curr Opin Hematol.

[10] Pereira J, Bronfman L, Bertín P, Marzouka E, Hidalgo P, Amaya S (1997). [Platelet alloimmunization in patients with oncologic blood disorders treated with multiple transfusions: prospective study in adults and children]. Rev Med Chil.

[11] Delaflor-Weiss E, Mintz PD (2000). The evaluation and management of platelet refractoriness and alloimmunization. Transfus Med Rev.

[12] Aster RH (1966). Pooling of platelets in the spleen: role in the pathogenesis of ‘hypersplenic’ thrombocytopenia. J Clin Invest.

[13] Densmore TL, Goodnough LT, Ali S, Dynis M, Chaplin H (1999). Prevalence of HLA sensitization in female apheresis donors. Transfusion.

[14] (1997). Leukocyte reduction and ultraviolet B irradiation of platelets to prevent alloimmunization and refractoriness to platelet transfusions. The Trial to Reduce Alloimmunization to Platelets Study Group. N Engl J Med.

[15] Vassallo RR (2007). New paradigms in the management of alloimmune refractoriness to platelet transfusions. Curr Opin Hematol.

